# Human Immunity and Susceptibility to Influenza A(H3) Viruses of Avian, Equine, and Swine Origin

**DOI:** 10.3201/eid2901.220943

**Published:** 2023-01

**Authors:** Elien Vandoorn, Wojciech Stadejek, Isabel Leroux-Roels, Geert Leroux-Roels, Anna Parys, Kristien Van Reeth

**Affiliations:** Ghent University, Merelbeke, Belgium (E. Vandoorn, W. Stadejek, A. Parys, K. Van Reeth);; Ghent University and Ghent University Hospital, Ghent, Belgium (I. Leroux-Roels, G. Leroux-Roels)

**Keywords:** Influenza A virus, humans, swine, birds, horses, hemagglutinins, antibodies, pandemics, public health, viruses, zoonoses, influenza, Belgium

## Abstract

Influenza A viruses (IAVs) of subtype H3 that infect humans are antigenically divergent from those of birds, horses, and swine. Human immunity against these viruses might be limited, implying potential pandemic risk. To determine human risk, we selected 4 avian, 1 equine, and 3 swine IAVs representing major H3 lineages. We tested serum collected during 2017–2018 from 286 persons in Belgium for hemagglutination inhibiting antibodies and virus neutralizing antibodies against those animal-origin IAVs and tested replication in human airway epithelia. Seroprevalence rates for circulating IAVs from swine in North America were >51%, swine in Europe 7%–37%, and birds and equids ≤12%. Replication was efficient for cluster IV-A IAVs from swine in North America and IAVs from swine in Europe, intermediate for IAVs from horses and poultry, and absent for IAVs from wild birds and a novel human-like swine IAV in North America. Public health risk may be highest for swine H3 IAVs.

Influenza A viruses (IAVs) of the H3 subtype are endemic to humans, swine, and wild birds; they also cause outbreaks in horses and are often detected in domestic birds. An H3 IAV that crosses the species barrier from animals to humans can result in a pandemic if the virus carries a hemagglutinin (HA) against which humans lack protective antibodies and the virus readily replicates in and spreads among humans. For example, in 1968, transmission of an IAV with an avian-origin H3 HA to humans caused the influenza A(H3N2) pandemic (*1*).

The natural IAV reservoir is considered to be wild waterfowl, but transmission to domestic poultry is frequent. Avian H3 IAVs are classified as Eurasian and North American lineages, although the HA of these viruses is antigenically closely related (*2*,*3*). In contrast, after being introduced to humans in 1968, the HA of human H3 IAVs quickly drifted away from that of the avian precursor IAV. Consequently, contemporary human H3 IAVs are antigenically divergent from those in birds (*2*). Similarly, avian H3 IAVs were introduced into horses in the 1960s, after which their HA antigenically drifted. That evolution was, however, different and slower than for human H3 IAVs (*4*). Equine H3 IAVs of Florida clade 1 (FC1) are currently predominant (*5*). All swine H3 IAVs derived their HA from human IAVs. 

H3 IAVs from swine in Europe originated from a human IAV that circulated in the late 1970s. Of the 2 major lineages cocirculating in North America, cluster IV-A was derived from human IAVs from the late 1990s and novel human-like swine H3 IAVs from human IAVs from the early 2010s (*6*). H3 IAVs undergo slower antigenic drift in swine than in humans. Consequently, persons born after the swine viruses’ human ancestor IAV had circulated are unlikely to have cross-reactive antibodies against the swine H3 IAVs. Therefore, with time, human population immunity against swine H3 IAVs decreases, increasing the pandemic risk (*7*–*10*).

The infectious potential of swine H3 IAVs for humans is evident from >400 recorded zoonotic infections in the United States caused by North American cluster IV-A or novel human-like H3 swine IAVs. Four zoonotic infections with H3 IAVs from swine in Europe have also been reported (*6*,*11*–*13*). H3 IAVs from equids can infect humans under experimental conditions, but there are no confirmed cases of natural transmission (*14*). Animal H3 IAVs might, however, become more adapted to humans by accumulating mutations in their viral proteins, reassortment of gene segments with IAVs of different species, or both (*6*,*15*). Avian H3 IAVs can infect humans directly or via an intermediate host, such as poultry or swine (*2*,*15*). In 2019, an H3N1 IAV that originated from wild birds caused outbreaks at 82 poultry farms in Belgium and 3 in France without infecting humans but was unusually virulent for poultry (*16*). In 2022, two zoonotic infections with avian H3N8 IAVs were reported (*17*).

H3 IAVs continue to evolve in each host species. Therefore, frequent re-evaluation of human seroprevalence and replication potential in humans for circulating animal H3 IAVs is recommended. Serum hemagglutination inhibiting (HI) and virus neutralizing (VN) antibodies correlate with protection. Thus, prevalence of such antibodies against animal H3 IAVs in persons of different age groups can be used to estimate the public health risk (*18*). Recent seroprevalence studies are available for H3 IAVs from swine in North America, but studies in Europe were conducted with samples and IAVs collected before 2011 (*7*–*10*,*19*). Studies for H3 IAVs from birds and equids are generally lacking, except for a few small-scale studies with historic IAV strains (*20*–*24*). The infectivity of animal H3 IAVs in humans was previously evaluated with mammalian models, outdated IAV strains, or both (*15*,*25*–*29*). To help evaluate the public health risk posed by different animal H3 IAVs, we analyzed serum samples collected from persons of different age groups in Belgium for prevalence and titers of HI and VN antibodies against all major circulating swine, avian, and equine H3 IAV lineages. We also assessed the replicative capacity of selected IAVs in human airway epithelia. The Commission for Medical Ethics of the Ghent University Hospital approved the study (approval no. 2017/834).

## Materials and Methods

### Human Serum and Tissue Samples

During August 2017–January 2018, we selected 286 serum samples from immunocompetent persons in Belgium born during 1921–2017 who had unknown influenza infection or vaccination history. The male:female ratio was ≈1:1, and we used ≈3 samples per birth year. 

From Epithelix Sàrl (https://www.epithelix.com), we purchased human airway epithelia (MucilAir) reconstituted from primary cells of biopsy samples from 6 donors (Table 1). We maintained the tissues at the air–liquid interface with MucilAir culture medium (Epithelix) according to the manufacturer’s instructions.

**Table 1:** Characteristics of MucilAir donors and tissues used for study of human susceptibility to influenza A(H3) viruses of avian, equine, and swine origin*

Donor ID	Donor no.	Age, y/sex	Ethnicity	Smoking status	Tissue type	Age of ALI at inoculation, wk	TEER ± SD, Ω.cm^2^†
ND1	MD0738	32/F	Caucasian	Nonsmoker	Nasal	11	774 ± 27
ND2	MD0436	46/M	Caucasian	Unknown	Nasal	11	1,256 ± 15
ND3	MD0722	61/M	Unknown	Unknown	Nasal	11	611 ± 27
BD1	MD0802	55/F	African	Nonsmoker	Bronchial	9	669 ± 32
BD2	MD0670	15/M	Caucasian	Nonsmoker	Bronchial	11	477 ± 23
BD3	MD0810	52/F	Hispanic	Nonsmoker	Bronchial	11	424 ± 24

*MucilAir, Epithelix Sàrl (https://www.epithelix.com). ALI, air–liquid interface; ID, identifier; TEER, transepithelial electrical resistance. †Average and SD on the basis of 12 values as mentioned in the certificate of analysis provided by the manufacturer, Epithelix.

### Viruses

**Figure 1:**
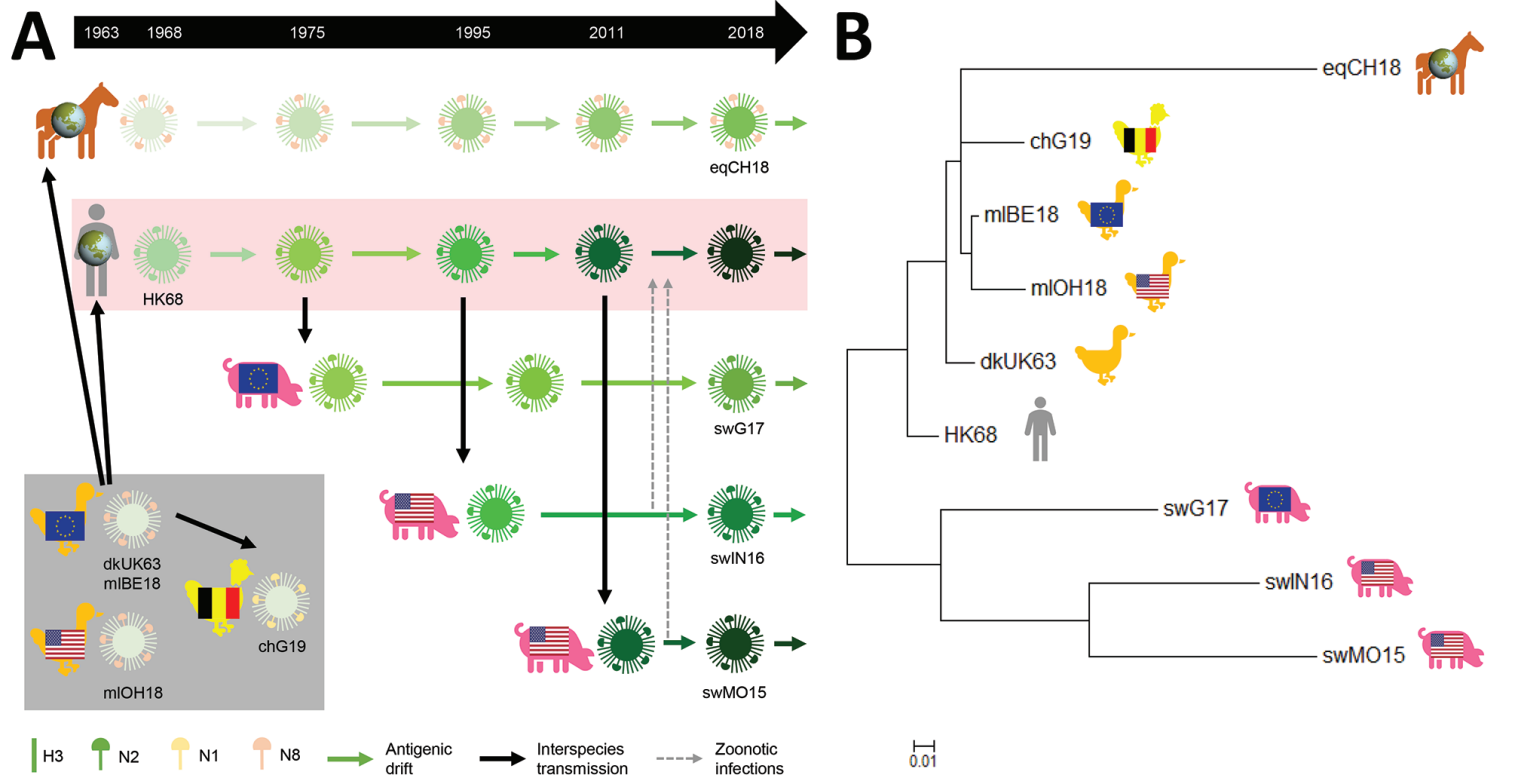
Epidemiologic and phylogenetic relationship between avian, equine, human, and swine influenza A test viruses. A) Schematic positioning of the test viruses in the influenza A(H3) virus epidemiology. B) Maximum-likelihood neighbor-joining phylogenetic tree of the hemagglutinin 1 of the test viruses. Complete isolate names are provided in Table 2. Scale bar indicates amino acid substitutions per site.

IAVs for seroreactivity and replication studies included representatives of major H3 lineages circulating in wild birds (mlOH18, mlBE18), horses (eqCH18), and swine (swG17, swIN16, swMO15); the avian H3N1 IAV that caused outbreaks in poultry in Belgium in 2019 (chG19); the 1968 human pandemic virus (HK68); and the presumed avian ancestor IAV of HK68 (dkUK63) (Table 2; Figure 1, panel A). We used epidemiologic data to select major H3 lineages (*5*,*30*–*32*). We selected test viruses on the basis of antigenic relatedness and amino acid homology to currently circulating IAVs of each lineage available in GenBank.

**Table 2:** IAV H3 strains used for study of human immunity and susceptibility to influenza A(H3) viruses of avian, equine, and swine origin*

Virus strain	Abbreviation	Subtype	Host	H3 lineage	H3 GenBank accession no.
A/duck/Ukraine/1/63	dkUK63	H3N8	Duck	Eurasian avian	HE802062
A/mallard/Ohio/18OS1219/2018	mlOH18	H3N8	Mallard	American avian	MN431078
A/*Anas platyrhynchos*/Belgium/7827/2018	mlBE18	H3N8	Mallard	Eurasian avian	MT407033
A/chicken/Belgium-Gent/136/2019	chG19	H3N1	Chicken	Eurasian avian	OP417305
A/equine/Chile/EQCL003/2018	eqCH18	H3N8	Horse	Equine Florida clade 1	OP467551
A/Hong Kong/1/68	HK68	H3N2	Human	Human pandemic	CY044261
A/swine/Gent/48/2017	swG17	H3N2	Pig	European swine	OP415564
A/swine/Indiana/A01729047/2016	swIN16	H3N2	Pig	N. Am. cluster IV-A swine	KU598305
A/swine/Missouri/A01840724/2015	swMO15	H3N2	Pig	N. Am. novel human-like swine	KP901306

*Horizontal rules represent grouping of IAVs isolated from similar host species; groups of host species are ordered chronologically according to the first detection of H3 IAVs in each of those species, and viruses of each species are ordered chronologically according to the time point at which the corresponding lineages first arose. IAV, influenza A virus; N. Am., North American.

The major target of neutralizing antibodies is HA1. We downloaded the viruses’ HA1 nucleotide sequences from GenBank and translated them to amino acids. We aligned sequences with the MUSCLE algorithm (https://www.ebi.ac.uk/Tools/msa/muscle) and constructed maximum-likelihood trees by using the Jones-Taylor-Thornton model and the nearest-neighbor-interchange heuristic method in MEGA7 (https://www.megasoftware.net) (*33*). We determined numbers of identical amino acids in presumed antigenic sites (*34*) and percentages of amino acid homology between test viruses by using MEGA7 and R version 3.5.3 (The R Foundation for Statistical Computing, https://www.r-project.org).

We received avian IAVs from the Flemish Institute for Biotechnology (Flanders, Belgium) and Ohio State University (Columbus, Ohio, USA), equine IAVs from St. Jude Children’s Research Hospital (Memphis, Tennessee, USA), North American swine IAVs from the US Department of Agriculture–Agricultural Research Service (Bethesda, Maryland, USA), and the human IAV from Philipps University Marburg (Marburg, Germany). Viruses for serologic assays and inoculation of MucilAir tissues were grown in MDCK cells; only avian and equine viruses for HI assays were propagated in allantoic cavities of 10-day-old embryonated chicken eggs; and all underwent <4 passages.

### Serologic Assays

HI and VN assays for antibodies against each test virus were performed according to standard procedures (*35*). We performed HI assays with 1% horse erythrocytes for avian and equine IAVs and 0.5% turkey erythrocytes for human and swine IAVs. Antibody titers represent the reciprocal of the highest serum dilution showing complete hemagglutination inhibition of 4 hemagglutinating units of virus (HI) or 50% neutralization of 100 tissue culture infective doses (TCID_50_) of virus (VN). Starting dilutions were 1:20 in HI and 1:10 in VN. We considered a titer of >40 to be positive.

### Virus Replication Kinetics

To standardize the amount of mucus, we washed the apical side of fully differentiated MucilAir tissues (1/donor/condition) 1 time with culture medium. Three days later, we inoculated the tissues apically with 250 μL of medium (mock inoculation) or IAV at multiplicity of infection 0.01 TCID_50_. After incubating the samples for 1 hour at 34°C and 5% CO_2_, we removed the inoculum and washed the apical side of the tissues 1 time. At 0–4 days postinoculation (dpi), we measured transepithelial electrical resistance (TEER) with a Millicell-ERS2 Voltohmmeter (Merck KGaA, https://www.merckmillipore.com) and took samples for virus titration. For titration, we added 250 μL medium apically, allowed it to equilibrate for 30 min at 34°C, and collected it. We determined TCID_50_ titers of inocula and samples by titration on MDCK monolayers, which 5 days later underwent immunocytochemical staining of IAV nucleoprotein, as previously described (*36*); the start dilution was 1:2.

### Statistical Analyses

We used log_2_-transformed antibody titers to calculate geometric mean titers (GMTs) and 95% CIs against each virus for samples from persons each birth decade. We used log_10_-transformed virus titers to calculate the area under the curve (AUC) for each virus in each MucilAir tissue. Samples that tested negative were assigned a titer of half the detection limit (HI 10, VN 5, virus titration 0.65 TCID_50_/mL). We used Kruskal-Wallis and Mann-Whitney U tests to compare antibody titers between viruses for a certain age group or between age groups for a certain virus. We used the same tests to compare AUCs between viruses for a certain tissue or between tissues for a certain virus. We compared proportions of positive samples by using Fisher exact tests. We determined Spearman correlation coefficients (CCs) between HI titers or between VN titers against different viruses by using nonstratified data. For serologic data, we applied the Bonferroni adjustment of the p values and considered adjusted p<0.05 significant. For AUCs, we considered p<0.1 significant. We performed all analyses with R version 3.5.3.

## Results

### Genetic Relatedness Between Test Viruses

For seroreactivity and replication studies in humans, we selected 9 H3 IAVs from humans, birds, horses, and swine. Their genetic relatedness was determined on the basis of HA1 amino acid sequence homology (Table 3; Figure 1). Human virus HK68 was closely related to the avian IAVs, showing 93%–96% homology and 32–34/40 identical amino acids in presumed antigenic sites. HK68 and avian IAVs were <83% homologous with recent equine and swine IAVs. Swine IAVs shared 24–26 aa in antigenic sites with HK68 and 17–23 aa with avian IAVs. Equine and swine IAVs were most distantly related; homology was <78% and 18–21 amino acids in antigenic sites. Whereas all avian IAVs were closely related, swine IAVs were antigenically distant from each other.

**Table 3:** Percentage amino acid homology (lower left) and number of identical amino acids out of 40 aa in presumed antigenic sites (upper right) (*34*) between hemagglutinin 1 of the H3 influenza A viruses used for study of human immunity and susceptibility to influenza A(H3) viruses of avian, equine, and swine origin*

Virus strain	Avian					Equine		Human		Swine		
	dkUK63	mlOH18	mlBE18	chG19		eqCH18		HK68†		swG17	swIN16	swMO15
	Eurasian	American	Eurasian	Eurasian		FC1		Pandemic		European	N. Am.	N. Am.
dkUK63		37	36	36		24		33		22	20	18
mlOH18	93.9		39	37		25		34		22	18	18
mlBE18	96.7	95.1		38		26		34		21	18	17
chG19	94.5	91.5	95.1			26		32		22	20	17
eqCH18	82.1	80.9	82.4	80.5				24		21	20	18
HK68	95.7	92.7	95.7	93.0		82.1				23	18	18
swG17	81.4	81.1	82.1	80.8		77.9		83.4			22	22
swIN16	80.8	78.7	80.8	80.8		73.2		81.4		79.4		26
swMO15	79.6	77.5	79.0	77.8		71.4		79.9		79.2	82.9	

*Viruses are ordered according to host species and chronologically according to the emergence of each of the influenza A virus lineages in these species. Blank cells indicate crossover points between comparisons. Complete isolate names are provided in Table 2. FC1, Florida clade 1; N. Am., North American. †Human influenza A virus that no longer circulates.

### Seroreactivity against HK68

**Figure 2:**
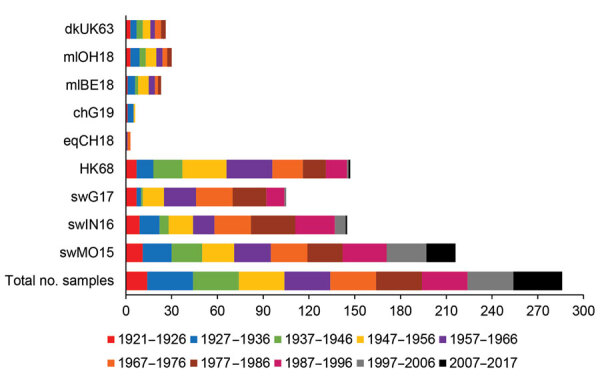
Number of positive human serum samples in the hemagglutination inhibition assay (titer >40) for each test virus compared with the total number of samples tested per birth cohort. Birth cohorts are represented by colors. A total of 286 serum samples collected during August 2017–January 2018 from immunocompetent persons in Belgium were tested. Complete isolate names are provided in Table 2.

**Figure 3:**
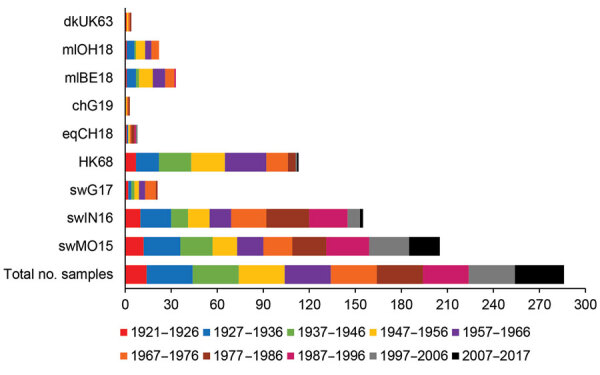
Number of positive human serum samples in the virus neutralization assay (titer >40) for each test virus compared with the total number of samples tested per birth cohort. Birth cohorts are represented by colors. A total of 286 serum samples collected during August 2017–January 2018 from immunocompetent persons in Belgium were tested. Complete isolate names are provided in Table 2.

When we tested human serum samples for antibodies against HK68 to evaluate potential exposure to this virus, we found that, overall, 51% were seropositive for HK68 in HI and 40% in VN assays (Figures 2, 3). Seroprevalence and GMTs were higher for persons born before 1977 (71% in HI, 65% in VN, GMTs >51) than for persons born during 1977–2017 (25% in HI, 6% in VN, GMTs <18; p<0.001) (Tables 4, 5). Seroreactivity was highest in those born during 1947–1966 and lowest in those born during 1997–2017.

**Table 4:** Geometric mean of hemagglutination inhibition antibody titers against H3 influenza A viruses of different species in different age groups, Belgium, 2017–2018*

Birth year range (age, y)†	No.	Avian					Equine		Human		Swine		
		dkUK63	mlOH18	mlBE18	chG19		eqCH18		HK68‡		swG17	swIN16	swMO15
		Eurasian	Am.	Eurasian	Eurasian		FC1		Pandemic		European	N. Am.	N. Am.
1921–1926 (96–91)	14	16 (10–25)	16 (10–23)	13 (10–19)	12 (10–16)		10 (10–10)		30 (17–52)		23 (14–38)	42 (24–75)	80 (29–219)
1927–1936 (90–81)	30	15 (12–19)	16 (12–21)	14 (11–16)	13 (10–16)		10 (10–11)		26 (20–35)		14 (11–18)	25 (17–36)	59 (35–99)
1937–1946 (80–71)	30	13 (11–16)	15 (12–19)	13 (11–15)	11 (10–12)		10 (10–10)		37 (27–51)		12 (11–14)	16 (12–22)	52 (32–83)
1947–1956 (70–61)	30	15 (12–19)	18 (14–23)	16 (12–21)	11 (10–13)		10 (10–10)		180 (128–252)		29 (21–39)	30 (20–43)	59 (39–91)
1957–1966 (60–51)	30	13 (11–16)	16 (13–20)	16 (13–20)	11 (10–12)		11 (10–13)		327 (249–432)		44 (30–63)	31 (22–44)	58 (40–83)
1967–1976 (50–41)	30	14 (11–18)	14 (11–17)	12 (10–15)	10 (10–11)		12 (10–15)		80 (43–150)		58 (40–83)	61 (40–91)	78 (49–124)
1977–1986 (40–31)	30	13 (10–17)	13 (10–15)	11 (10–14)	10 (10–11)		10 (10–11)		32 (21–50)		45 (34–58)	146 (105–202)	98 (60–162)
1987–1996 (30–21)	30	10 (10–11)	10 (10–10)	10 (10–10)	10 (10–10)		10 (10–10)		30 (21–42)		25 (18–35)	139 (87–224)	254 (170–380)
1997–2006 (20–11)	30	10 (10–10)	10 (10–10)	10 (10–10)	10 (10–10)		10 (10–10)		10 (10–12)		11 (10–13)	19 (14–26)	101 (64–158)
2007–2017 (10–0)	32	10 (10–10)	10 (10–10)	10 (10–10)	10 (10–10)		10 (10–10)		11 (10–12)		11 (10–13)	11 (10–13)	37 (25–54)
1921–2017 (96–0)	286	13 (12–13)	13 (12–14)	12 (12–13)	11 (10–11)		10 (10–11)		42 (36–49)		23 (21–26)	36 (31–42)	75 (64–87)

*Values represent geometric mean hemagglutination inhibition titers (95% CI). Viruses are ordered according to host species and chronologically according to the emergence of each of the influenza A virus lineages in these species. Complete isolate names are provided in Table 2. Am., American; FC1, Florida clade 1; N. Am., North American. †Age at the end of 2017. ‡Human influenza A virus that no longer circulates.

**Table 5:** Geometric mean of virus neutralization antibody titers against H3 influenza A viruses of different species in different age groups of the human population, 2017–2018, Belgium*

Birth year range (age, y)†	No.	Avian					Equine		Human		Swine		
		dkUK63	mlOH18	mlBE18	chG19		eqCH18		HK68‡		swG17	swIN16	swMO15
		Eurasian	Am.	Eurasian	Eurasian		FC1		Pandemic		European	N. Am.	N. Am.
1921–1926 (96–91)	14	7 (5–9)	10 (7–15)	8 (6–12)	6 (5–7)		7 (5–10)		30 (12–72)		10 (5–19)	104 (44–247)	116 (38–351)
1927–1936 (90–81)	30	6 (5–7)	12 (9–18)	12 (8–17)	6 (5–7)		7 (5–8)		43 (27–68)		9 (6–11)	60 (34–106)	105 (62–176)
1937–1946 (80–71)	30	5 (5–6)	9 (7–12)	10 (7–14)	5 (5–6)		6 (5–6)		48 (32–72)		9 (6–12)	33 (21–53)	79 (46–137)
1947–1956 (70–61)	30	7 (5–9)	14 (10–21)	15 (11–22)	6 (5–7)		7 (5–9)		69 (44–110)		8 (6–11)	31 (18–52)	35 (20–60)
1957–1966 (60–51)	30	6 (5–7)	15 (11–20)	19 (13–27)	7 (5–9)		6 (5–6)		100 (72–138)		12 (8–16)	28 (17–47)	37 (24–56)
1967–1976 (50–41)	30	7 (5–9)	11 (8–17)	12 (8–17)	6 (5–7)		6 (5–7)		31 (17–55)		18 (13–25)	94 (56–157)	60 (35–103)
1977–1986 (40–31)	30	6 (5–7)	7 (6–8)	6 (5–7)	5 (5–6)		8 (6–10)		9 (6–13)		9 (7–12)	285 (175–462)	84 (45–154)
1987–1996 (30–21)	30	5 (5–5)	6 (5–6)	5 (5–6)	5 (5–5)		6 (5–7)		6 (5–6)		7 (6–8)	219 (129–372)	225 (156–323)
1997–2006 (20–11)	30	5 (5–5)	5 (5–5)	5 (5–5)	5 (5–5)		5 (5–6)		5 (5–6)		6 (5–7)	18 (11–29)	163 (96–275)
2007–2017 (10–0)	32	5 (5–5)	5 (5–5)	5 (5–5)	5 (5–5)		6 (5–7)		6 (5–7)		6 (5–6)	8 (6–10)	40 (24–67)
1921–2017 (96–0)	286	6 (5–6)	9 (8–10)	9 (8–10)	6 (5–6)		6 (6–7)		21 (18–25)		9 (8–9)	49 (40–60)	77 (64–91)

*Values represent geometric mean virus neutralization titers (95% CI). Viruses are ordered according to host species and chronologically according to the emergence of each of the influenza A virus lineages in these species. Complete isolate names are provided in Table 2. Am., American; FC1, Florida clade 1; N. Am., North American. †Age at the end of 2017. ‡Human influenza A virus that no longer circulates.

### Seroreactivity against Avian H3 IAVs

DkUK63 is the presumed avian ancestor IAV of HK68. MlOH18 and mlBE18 represent North American and Eurasian lineage H3 IAVs currently circulating in wild birds. For these IAVs, <10% were seropositive in HI and <12% in VN (Figures 2, 3). Differences in seroprevalence rates between the 3 IAVs for each age group or between age groups for each IAV were not significant, except VN seroprevalence for mlBE18 of persons born during 1947–1956 (9%) and those born during 2007–2017 (0%; p<0.04). GMTs were <20 for all age groups, and no HI or VN antibodies against the 3 IAVs of wild birds were detected in persons born during 1997–2017 (Tables 4, 5).

ChG19 represents the avian H3N1 IAV that caused outbreaks in poultry in Belgium during 2019. Overall seroprevalence rates for chG19 were 2% in HI and 1% in VN (Figures 2, 3), and differences in seroprevalence rates between age groups were not significant. GMTs were below the detection limit for all age groups and no antibodies against chG19 were detected in persons born during 1987–2017 (Tables 4, 5).

### Seroreactivity Against Equine H3 IAVs

The predominant H3 IAVs in horses belong to FC1, represented by eqCH18. Only 1% of all serum samples tested positive against eqCH18 in HI and 3% in VN (Figures 2, 3). GMTs were below the detection limit for all age groups (Tables 4, 5). Seroreactivity against eqCH18 did not differ significantly between age groups.

### Seroreactivity against Swine H3 IAVs

SwG17 represents contemporary H3 IAVs in swine in Europe. Of all persons tested, 37% were seropositive for swG17 in HI and 7% in VN (Figures 2, 3). Seroprevalence rates and GMTs were higher among persons born before 1997 (HI 46%, VN 9%, GMTs >28) than among persons born during 1997–2017 (HI 2%, VN 0%, GMTs ≤11; p<0.02) and peaked among those born during 1967–1976 (Tables 4, 5).

The 2 predominant H3 IAV lineages currently circulating among swine in North America are North American cluster IV-A (represented by swIN16) and novel human-like swine IAVs (represented by swMO15). At least half of all serum samples tested positive for swIN16, 51% in HI and 54% in VN (Figures 2, 3). Seroprevalence rates and GMTs were higher among persons born before 1997 (61% in HI, 65% in VN, GMTs >46) than for those born during 1997–2017 (13% in HI, 16% in VN, GMTs <14; p<0.001) and peaked among persons born during 1977–1986 (Tables 4, 5).

Overall seroprevalence rates for swMO15 were 76% in HI and 72% in VN (Figures 2, 3). At least 50% of persons in each age group were positive in both HI and VN, with GMTs of ≥35 (Tables 4, 5). Seroreactivity was highest for persons born during 1987–1996, and significant differences in seroprevalence were found only between those in this group and those born during 2006–2017 in HI (97% vs. 59%; p = 0.02) and those born during 1947–1956 in VN (93% vs. 53%; p = 0.04). Seroreactivity was higher against IAVs of the swine H3 lineages that were more recently introduced to swine and peaked among persons born shortly before these introductions.

### Correlations between Antibody Titers against Different H3 IAVs

Antibody titers against avian IAVs were highly correlated (CC = 0.39–0.85 in HI, CC = 0.47–0.85 in VN) (Table 6). Titers against HK68 were highly correlated with those against IAVs of wild birds (CC = 0.45–0.50 in HI, CC = 0.48–0.72 in VN). CCs between titers against other epidemiologically related IAVs of different species (dkUK63 and eqCH18, HK68 and swG17) were lower (CC = 0.28–0.58 in HI, CC = 0.22–0.29 in VN). Titers against different swine IAVs showed variable CCs (0.35–0.61 in HI and 0.14–0.50 in VN [the first value of which is not significant]).

**Table 6:** Spearman correlation coefficients between hemagglutination inhibition antibody titers against influenza A(H3) viruses of different species (upper right) and between virus neutralization antibody titers against H3 influenza A viruses of different species (lower left)*

Virus strain	Avian					Equine		Human		Swine		
	dkUK63	mlOH18	mlBE18	chG19		eqCH18		HK68†		swG17	swIN16	swMO15
	Eurasian	American	Eurasian	Eurasian		FC1		Pandemic		European	N. Am.	N. Am.
dkUK63		0.76	0.73	0.39		0.28		0.45		0.27	NS	NS
mlOH18	0.49		0.85	0.45		0.28		0.49		0.27	NS	NS
mlBE18	0.49	0.85		0.46		0.33		0.50		0.28	NS	NS
chG19	0.55	0.47	0.49			0.25		0.22		NS	NS	NS
eqCH18	0.22	NS	NS	0.27				0.27		0.21	NS	NS
HK68	0.48	0.66	0.72	0.44		NS				0.58	0.26	NS
swG17	NS	0.25	0.28	0.22		NS		0.29			0.61	0.35
swIN16	NS	NS	NS	NS		NS		NS		0.38		0.60
swMO15	NS	NS	NS	NS		NS		NS		NS	0.50	

*Viruses are ordered according to host species and chronologically according to the emergence of each of the influenza A virus (IAV) lineages in these species. Blank cells indicate crossover points between comparisons. Complete isolate names are provided in Table 2. FC1, Florida clade 1; N. Am., North American; NS, not significant. †Human IAV that no longer circulates.

### Replication Kinetics of H3 IAVs in Human Airway Epithelia

**Figure 4:**
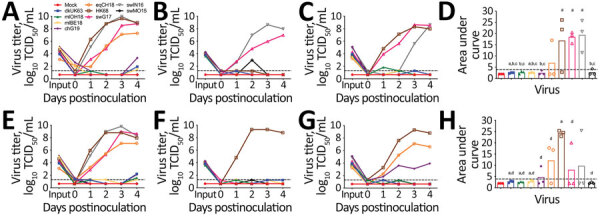
Replication kinetics of influenza A(H3) viruses of different species in human airway epithelia (MucilAir; Epithelix Sàrl, https://www.epithelix.com). Tissues were infected with viruses at a multiplicity of infection of 0.01 TCID_50_, and supernatants were collected at different days postinfection for virus titration in MDCK cells. A–C) Virus replication in nasal tissue of donors ND1 (A), ND2 (B), and ND3 (C). D) Virus yield in nasal tissues. E–G) Virus replication in bronchial tissue of donors BD1 (E), BD2 (F), and BD3 (G). H) Virus yield in bronchial tissues. Virus yield in panels D and H was determined by calculating the area under the curve at 1–4 dpi; letters indicate significant differences (p<0.1): mock (a), swG17 (b), swIN16 (c), or HK68 (d). Black dashed lines represent detection limit. Complete isolate names are provided in Table 2. TCID_50_, 50% tissue culture infectious dose.

**Figure 5:**
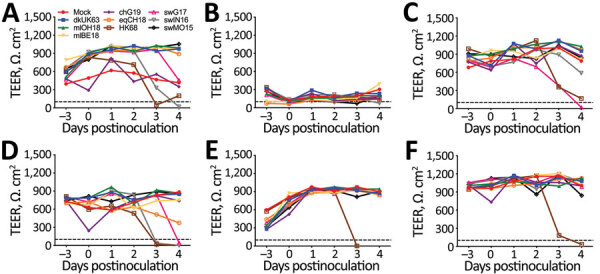
TEER of human airway epithelia (MucilAir; Epithelix Sàrl, https://www.epithelix.com) at different days postinfection with influenza A(H3) viruses of different species at a multiplicity of infection of 0.01 50% tissue culture infective dose. TEER is shown for nasal tissue of donors ND1 (A), ND2 (B), and ND3 (C) and for bronchial tissue of donors BD1 (E), BD2 (F), and BD3 (G). Black dashed lines represent the TEER below which tissue integrity is irreversibly lost (*37*). Complete isolate names are provided in Table 2. TEER, transepithelial electrical resistance.

Human HK68 virus replicated to titers of up to 9.6 log_10_ TCID_50_/mL in all human airway epithelia except that of ND2 (Figure 4). Similar high titers of cluster IV-A H3 IAV swIN16 from swine in North America and swG17 from swine in Europe were detected in all nasal tissues and tissues of BD1 (Figure 4). Replication of these 3 IAVs peaked at 2–4 dpi and generally caused irreversible tissue damage, indicated by a decrease in TEER to values of <100 Ω.cm^2^ (*37*) (Figure 5).

Equine IAV eqCH18 replicated efficiently in tissues of ND1, BD1, and BD3 without considerably affecting TEER (Figures 4, 5). Peak titers were detected at 3–4 dpi and were 1.5–2.7 log_10_ TCID_50_/mL lower than those for HK68, swIN16, and swG17.

ChG19 isolated from poultry replicated to titers of up to 3.9 log_10_ TCID_50_/mL in bronchial tissue of BD3 without affecting TEER (Figures 4, 5). ChG19 was also detectable in tissues of ND1 and BD1 at 4 dpi.

For the 3 IAVs of wild birds and North American novel human-like swine IAV swMO15, no virus was detectable in any of the tissues except dkUK63, mlOH18, and mlB18 had titers of <2.2 log_10_ TCID_50_/mL at 4 dpi in tissues of ND1 and BD1, and an swMO15 titer of 3.0 log_10_ TCID_50_/mL was detected at 2 dpi in the tissue of ND2 (Figures 4, 5). Because of large donor-to-donor variation, only a few differences in virus replication AUCs were significant (p<0.1). In nasal tissues, AUCs were significantly higher for swG17 and swIN16 than for all avian IAVs and swMO15. In bronchial tissues, AUCs were significantly higher for HK68 than for all avian IAVs, eqCH18, and swMO15.

## Discussion

Antibody titers against animal H3 IAVs in serum samples from humans in Belgium depended on the virus strain and the person’s birth year. Overall seroprevalence rates were high (>51%) for IAVs from swine in North America, intermediate (7%–37%) for IAVs from swine in Europe, and low (≤12%) for IAVs from birds and equids. Seroreactivity against swine IAVs was highest among persons born during 1967–1996, and seroreactivity against almost all IAVs was lowest among the youngest persons, born during 1997–2017. These results are consistent with findings of previous studies with other, often older, swine IAV strains and studies that tested only a low number of serum samples from adults against historic avian or equine IAVs (*7*–*10*,*19*–*24*,*31*,*38*). Cluster IV-A IAVs from swine in North America and H3 IAVs from swine in Europe replicated efficiently in human airway epithelia, whereas replication was intermediate for H3 IAVs of horses and poultry, and minimal for H3 IAVs of wild birds and a North American novel human-like swine H3 IAV. Our results for cluster IV-A IAVs from swine in North America and IAVs from swine in Europe are consistent with previous findings in differentiated human (tracheo)bronchial epithelial cells (*25*,*27*). However, 1 study also reported efficient replication of a zoonotic novel human-like IAV from swine in North America (*27*). A previous study with historic strains detected substantial replication of avian H3 IAVs, whereas an equine H3 IAV did not replicate (*25*). Discrepancies between our findings and previous findings can result from the use of different cell systems and variation in the genetic background of human cell donors or IAV strains (*39*,*40*).

**Figure 6:**
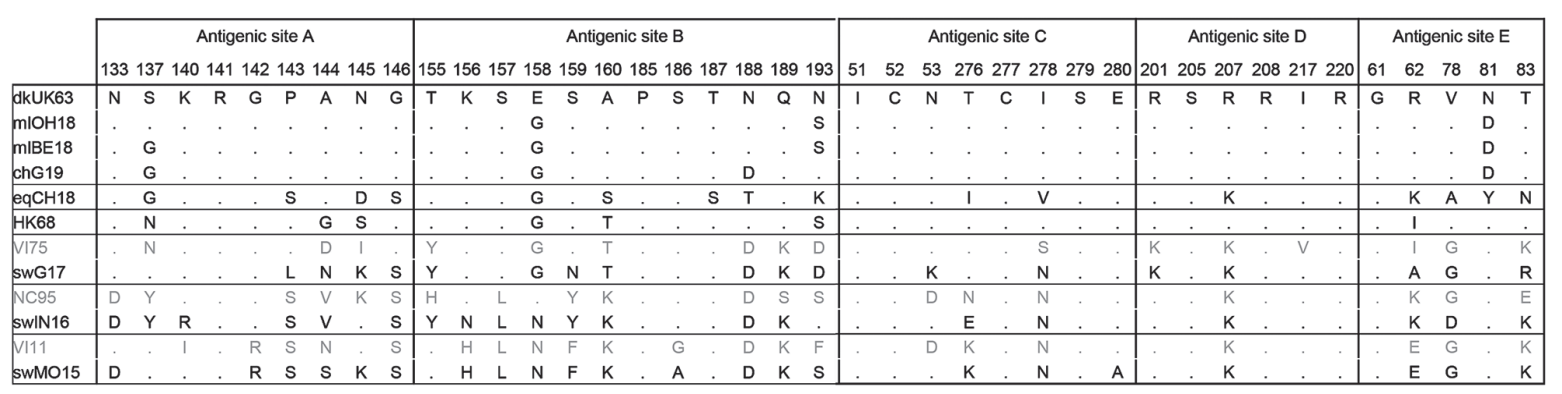
Amino acids at presumed antigenic sites of the hemagglutinin 1 (*34*) of the influenza A(H3) viruses used in this study and their presumed ancestor viruses. Dots indicate that the amino acid is the same as that for dkUK63. Gray indicates the presumed human ancestor viruses of swG17 (A/Victoria/3/75 [VI75]), swIN16 (A/Nanchang/933/95 [NC95]), and swMO15 (A/Victoria/361/2011 [VI11]), which were not included as test viruses in this study. Complete isolate names are provided in Table 2.

Antibody titers against swine H3 IAVs reflect cross-reactive titers against human ancestor IAVs. Antibodies against human ancestor IAVs can be deduced from the theory of antigenic seniority: humans are likely to have antibodies against human IAVs that circulated after their birth, with peak titers against IAVs encountered early in life (*41*), confirmed by our results for HK68. Our findings, together with those of previous studies showing similar seroreactivity against older swine H3 IAVs and their human ancestor IAV (*7*,*9*,*10*), suggest slow antigenic drift of H3 HA in swine and indicate swine as a reservoir for historic human IAVs. We estimate that HA1 amino acid homology between our swine test viruses and their human ancestor is 87%–93%, with 29–32 identical amino acids in antigenic sites (Figure 6). Although the HA1 sequences of the avian H3 IAVs were more closely related to that of human virus HK68, cross-reactive serum antibody titers were minimal. Accordingly, ferret serum against human H3 IAVs showed low cross-reactivity with avian H3 IAVs (*42*), which might be caused by a few key amino acid differences between HK68 and avian H3 IAVs. Compared with all avian IAVs, HK68 has 4 mutations in antigenic sites, of which N145S might be of particular relevance. This mutation mediated antigenic cluster transitions for swine and human H3 IAVs (*43*,*44*). Furthermore, higher HA glycosylation of human IAVs might mask certain epitopes shared with avian IAVs, preventing humans from raising antibodies against these epitopes. For example, glycosylation at positions 122, 133, and 144 masks epitopes in antigenic site A. In contrast, HA glycosylation patterns for swine IAVs and for the human ancestor IAV are similar (*45*). For equine H3 IAVs, the lack of cross-reactive antibodies can be explained by the closer relatedness to avian than to human IAVs and substantial antigenic drift in horses after the introduction from the avian reservoir (*2*,*3*).

Seroprevalence rates of <12% for avian and equine H3 IAVs suggest that these IAVs pose a high pandemic risk. Comparable seroprevalences of 2%–19% against the 2009 pandemic influenza A(H1N1) virus were detected right before the pandemic started (*46*). However, more efficient replication of H3 IAVs of swine in human respiratory tissues as opposed to those of birds or horses suggests that swine pose the highest risk for introduction of H3 IAVs to humans. Indeed, 434 human infections with cluster IV-A and novel human-like H3 IAVs from swine in North America and 4 infections with H3 IAVs from swine in Europe have been reported (*11*–*13*), whereas only 2 zoonotic infections with avian H3 IAVs and no zoonotic infections with equine H3 IAVs have been reported. Swine IAVs are derived from past human IAVs, which can explain their higher potential to infect humans. Swine IAVs prefer human-type α-2,6 sialic acid receptors, whereas avian and equine IAVs prefer avian-type α-2,3 receptors. Human cells also support polymerase activity of swine but not avian IAVs (*47*). In addition, humans frequently encounter dense swine populations and, unlike for horses and poultry, H3 IAVs are endemic among swine. Because zoonotic infections generally result from close contact with infected animals, swine IAVs are also more likely than IAVs of horses or birds to be transmitted to humans (*48*). On the basis of the seroprevalence rates of >30% for persons >16 years of age, swine H3 IAVs are considered a lower pandemic risk (*18*). They do, however, pose a zoonotic risk to the youngest persons who lack cross-reactive antibodies, which can explain why most human infections with swine H3 IAVs occurred in persons <18 years of age (*11*–*13*). Our results suggest that population immunity will wane over time and that the human population will sooner become fully susceptible to H3 IAVs from swine in Europe than to H3 IAVs from swine in North America.

We estimated the infection potential of animal H3 IAVs in humans on the basis of their replicative capacity in nasal and bronchial MucilAir tissues. However, adaptive and some innate immune responses that are not represented in this model might cause more restricted replication of swine, equine, or avian H3 IAVs in vivo. Also, in vitro experiments in differentiated human airway epithelia will, in the best case, reflect only replication efficiency in a single person and are in no way indicative of airborne transmission between humans (*49*). 

In conclusion, our results stress the need to closely monitor circulating H3 IAVs in different animal species and to frequently evaluate humans for antibodies against these IAVs. This need applies especially to H3 IAVs of swine, which seem to pose the highest zoonotic risk.

## References

[1] Fang R, Min Jou W, Huylebroeck D, Devos R, Fiers W. Complete structure of A/duck/Ukraine/63 influenza hemagglutinin gene: animal virus as progenitor of human H3 Hong Kong 1968 influenza hemagglutinin. Cell. 1981;25:315–23. 6169439 10.1016/0092-8674(81)90049-0

[2] Bailey E, Long LP, Zhao N, Hall JS, Baroch JA, Nolting J, et al. Antigenic characterization of H3 subtypes of avian influenza A viruses from North America. Avian Dis. 2016;60(Suppl):346–53. 27309078 10.1637/11086-041015-RegR PMC4911812

[3] Pu J, Liu QF, Xia YJ, Fan YL, Brown EG, Tian FL, et al. Genetic analysis of H3 subtype influenza viruses isolated from domestic ducks in northern China during 2004–2005. Virus Genes. 2009;38:136–42. 19067150 10.1007/s11262-008-0300-7

[4] Woodward A, Rash AS, Medcalf E, Bryant NA, Elton DM. Using epidemics to map H3 equine influenza virus determinants of antigenicity. Virology. 2015;481:187–98. 25797606 10.1016/j.virol.2015.02.027

[5] World Organisation for Animal Health. OIE expert surveillance panel on equine influenza vaccine composition. 2020 Apr 16 [cited 2021 Aug 27]. https://oiebulletin.fr/?officiel=08-4-2-2020-1-panel

[6] Anderson TK, Chang J, Arendsee ZW, Venkatesh D, Souza CK, Kimble JB, et al. Swine influenza A viruses and the tangled relationship with humans. Cold Spring Harb Perspect Med. 2021;11:a038737. 31988203 10.1101/cshperspect.a038737 PMC7919397

[7] Qiu Y, Muller CP, Van Reeth K. Lower seroreactivity to European than to North American H3N2 swine influenza viruses in humans, Luxembourg, 2010. Euro Surveill. 2015;20:25–33. 25860393 10.2807/1560-7917.es2015.20.13.21078

[8] Lorbach JN, Fitzgerald T, Nolan C, Nolting JM, Treanor JJ, Topham DJ, et al. Gaps in serologic immunity against contemporary swine-origin influenza A viruses among healthy individuals in the United States. Viruses. 2021;13:127. 33477472 10.3390/v13010127 PMC7830885

[9] Krumbholz A, Lange J, Dürrwald R, Walther M, Müller TH, Kühnel D, et al. Prevalence of antibodies to European porcine influenza viruses in humans living in high pig density areas of Germany. Med Microbiol Immunol (Berl). 2014;203:13–24. 24013183 10.1007/s00430-013-0309-y

[10] Hoschler K, Thompson C, Casas I, Ellis J, Galiano M, Andrews N, et al. Population susceptibility to North American and Eurasian swine influenza viruses in England, at three time points between 2004 and 2011. Euro Surveill. 2013;18:20578. 24079379 10.2807/1560-7917.es2013.18.36.20578

[11] US Centers for Disease Control and Prevention. Novel influenza A virus infections [cited 2021 Nov 21]. https://gis.cdc.gov/grasp/fluview/Novel_Influenza.html

[12] Freidl GS, Meijer A, de Bruin E, de Nardi M, Munoz O, Capua I, et al. FLURISK Consortium. Influenza at the animal-human interface: a review of the literature for virological evidence of human infection with swine or avian influenza viruses other than A(H5N1). Euro Surveill. 2014;19:20793. 24832117 10.2807/1560-7917.es2014.19.18.20793

[13] Piralla A, Moreno A, Orlandi ME, Percivalle E, Chiapponi C, Vezzoli F, et al.; Influenza Surveillance Study Group. Swine influenza A(H3N2) virus infection in immunocompromised man, Italy, 2014. Emerg Infect Dis. 2015;21:1189–91. 26079745 10.3201/eid2107.140981 PMC4480377

[14] Xie T, Anderson BD, Daramragchaa U, Chuluunbaatar M, Gray GC. A review of evidence that equine influenza viruses are zoonotic. Pathogens. 2016;5:3–10. 27420100 10.3390/pathogens5030050 PMC5039430

[15] Baz M, Paskel M, Matsuoka Y, Zengel J, Cheng X, Jin H, et al. Replication and immunogenicity of swine, equine, and avian H3 subtype influenza viruses in mice and ferrets. J Virol. 2013;87:6901–10. 23576512 10.1128/JVI.03520-12 PMC3676140

[16] Steensels M, Gelaude P, Van Borm S, Van Den Berg T, Cargnel M, Roupie V, et al. Atypical pathogenicity of avian influenza (H3N1) virus involved in outbreak, Belgium, 2019. Emerg Infect Dis. 2020;26:1899–903. 32687049 10.3201/eid2608.191338 PMC7392414

[17] Tan X, Yan X, Liu Y, Wu Y, Liu JY, Mu M, et al. A case of human infection by H3N8 influenza virus. Emerg Microbes Infect. 2022;11:2214–7. 36000153 10.1080/22221751.2022.2117097 PMC9542523

[18] World Health Organization. Tool for Influenza Pandemic Risk Assessment (TIPRA) version 1. 2016 May [cited 2021 Sept 28]. https://apps.who.int/iris/handle/10665/250130

[19] Fragaszy E, Ishola DA, Brown IH, Enstone J, Nguyen-Van-Tam JS, Simons R, et al.; Flu Watch Group. Combating Swine Influenza (COSI) Consortium. Increased risk of A(H1N1)pdm09 influenza infection in UK pig industry workers compared to a general population cohort. Influenza Other Respir Viruses. 2016;10:291–300. 26611769 10.1111/irv.12364 PMC4910179

[20] Sikkema RS, Freidl GS, de Bruin E, Koopmans M. Weighing serological evidence of human exposure to animal influenza viruses - a literature review. Euro Surveill. 2016;21:30388. 27874827 10.2807/1560-7917.ES.2016.21.44.30388 PMC5114483

[21] Zhou N, He S, Zhang T, Zou W, Shu L, Sharp GB, et al. Influenza infection in humans and pigs in southeastern China. Arch Virol. 1996;141:649–61. 8645101 10.1007/BF01718323

[22] Burnell FJ, Holmes MA, Roiko AH, Lowe JB, Heil GL, White SK, et al. Little evidence of human infection with equine influenza during the 2007 epizootic, Queensland, Australia. J Clin Virol. 2014;59:100–3. 24360918 10.1016/j.jcv.2013.11.011

[23] Khurelbaatar N, Krueger WS, Heil GL, Darmaa B, Ulziimaa D, Tserennorov D, et al. Little evidence of avian or equine influenza virus infection among a cohort of Mongolian adults with animal exposures, 2010-2011. PLoS One. 2014;9:e85616. 24465622 10.1371/journal.pone.0085616 PMC3897462

[24] Larson KR, Heil GL, Chambers TM, Capuano A, White SK, Gray GC. Serological evidence of equine influenza infections among persons with horse exposure, Iowa. J Clin Virol. 2015;67:78–83. 25959164 10.1016/j.jcv.2015.04.009

[25] Ilyushina NA, Ikizler MR, Kawaoka Y, Rudenko LG, Treanor JJ, Subbarao K, et al. Comparative study of influenza virus replication in MDCK cells and in primary cells derived from adenoids and airway epithelium. J Virol. 2012;86:11725–34. 22915797 10.1128/JVI.01477-12 PMC3486302

[26] Shin DL, Yang W, Peng JY, Sawatsky B, von Messling V, Herrler G, et al. Avian influenza A virus infects swine airway epithelial cells without prior adaptation. Viruses. 2020;12:589. 32481674 10.3390/v12060589 PMC7374723

[27] Sun X, Pulit-Penaloza JA, Belser JA, Pappas C, Pearce MB, Brock N, et al. Pathogenesis and transmission of genetically diverse swine-origin H3N2 variant influenza A viruses from multiple lineages isolated in the United States, 2011–2016. J Virol. 2018;92:e00665–18. 29848587 10.1128/JVI.00665-18 PMC6069210

[28] Driskell EA, Jones CA, Stallknecht DE, Howerth EW, Tompkins SM. Avian influenza virus isolates from wild birds replicate and cause disease in a mouse model of infection. Virology. 2010;399:280–9. 20123144 10.1016/j.virol.2010.01.005

[29] Solórzano A, Foni E, Córdoba L, Baratelli M, Razzuoli E, Bilato D, et al. Cross-species infectivity of H3N8 influenza virus in an experimental infection in swine. J Virol. 2015;89:11190–202. 26311894 10.1128/JVI.01509-15 PMC4645675

[30] Walia RR, Anderson TK, Vincent AL. Regional patterns of genetic diversity in swine influenza A viruses in the United States from 2010 to 2016. Influenza Other Respir Viruses. 2019;13:262–73. 29624873 10.1111/irv.12559 PMC6468071

[31] Henritzi D, Petric PP, Lewis NS, Graaf A, Pessia A, Starick E, et al. Surveillance of European domestic pig populations identifies an emerging reservoir of potentially zoonotic swine influenza A viruses. Cell Host Microbe. 2020;28:614–627.e6. 32721380 10.1016/j.chom.2020.07.006

[32] Choi JG, Kang HM, Kim MC, Paek MR, Kim HR, Kim BS, et al. Genetic relationship of H3 subtype avian influenza viruses isolated from domestic ducks and wild birds in Korea and their pathogenic potential in chickens and ducks. Vet Microbiol. 2012;155:147–57. 21955449 10.1016/j.vetmic.2011.08.028

[33] Kumar S, Stecher G, Tamura K. MEGA7: Molecular Evolutionary Genetics Analysis version 7.0 for bigger datasets. Mol Biol Evol. 2016;33:1870–4. 27004904 10.1093/molbev/msw054 PMC8210823

[34] Rajão DS, Gauger PC, Anderson TK, Lewis NS, Abente EJ, Killian ML, et al. Novel reassortant human-like H3N2 and H3N1 influenza A viruses detected in pigs are virulent and antigenically distinct from swine viruses endemic to the United States. J Virol. 2015;89:11213–22. 26311895 10.1128/JVI.01675-15 PMC4645639

[35] Van Reeth K, Gregory V, Hay A, Pensaert M. Protection against a European H1N2 swine influenza virus in pigs previously infected with H1N1 and/or H3N2 subtypes. Vaccine. 2003;21:1375–81. 12615433 10.1016/s0264-410x(02)00688-6

[36] Van Poucke SG, Nicholls JM, Nauwynck HJ, Van Reeth K. Replication of avian, human and swine influenza viruses in porcine respiratory explants and association with sialic acid distribution. Virol J. 2010;7:38. 20158900 10.1186/1743-422X-7-38 PMC2829537

[37] Boda B, Benaoudia S, Huang S, Bonfante R, Wiszniewski L, Tseligka ED, et al. Antiviral drug screening by assessing epithelial functions and innate immune responses in human 3D airway epithelium model. Antiviral Res. 2018;156:72–9. 29890184 10.1016/j.antiviral.2018.06.007 PMC7113743

[38] Liu F, Levine MZ. Heterologous antibody responses conferred by A(H3N2) variant and seasonal influenza vaccination against newly emerged 2016–2018 A(H3N2) variant viruses in healthy persons. Clin Infect Dis. 2020;71:3061–70. 31858129 10.1093/cid/ciz1203 PMC8974393

[39] Ciminski K, Chase GP, Beer M, Schwemmle M. Influenza A viruses: understanding human host determinants. Trends Mol Med. 2021;27:104–12. 33097424 10.1016/j.molmed.2020.09.014

[40] Stewart CE, Torr EE, Mohd Jamili NH, Bosquillon C, Sayers I. Evaluation of differentiated human bronchial epithelial cell culture systems for asthma research. J Allergy (Cairo). 2012;2012:943982. 22287976 10.1155/2012/943982 PMC3263641

[41] Fonville JM, Wilks SH, James SL, Fox A, Ventresca M, Aban M, et al. Antibody landscapes after influenza virus infection or vaccination. Science. 2014;346:996–1000. 25414313 10.1126/science.1256427 PMC4246172

[42] Guan L, Shi J, Kong X, Ma S, Zhang Y, Yin X, et al. H3N2 avian influenza viruses detected in live poultry markets in China bind to human-type receptors and transmit in guinea pigs and ferrets. Emerg Microbes Infect. 2019;8:1280–90. 31495283 10.1080/22221751.2019.1660590 PMC6746299

[43] Smith DJ, Lapedes AS, de Jong JC, Bestebroer TM, Rimmelzwaan GF, Osterhaus AD, et al. Mapping the antigenic and genetic evolution of influenza virus. Science. 2004;305:371–6. 15218094 10.1126/science.1097211

[44] de Jong JC, Smith DJ, Lapedes AS, Donatelli I, Campitelli L, Barigazzi G, et al. Antigenic and genetic evolution of swine influenza A (H3N2) viruses in Europe. J Virol. 2007;81:4315–22. 17287258 10.1128/JVI.02458-06 PMC1866135

[45] Tharakaraman K, Raman R, Stebbins NW, Viswanathan K, Sasisekharan V, Sasisekharan R. Antigenically intact hemagglutinin in circulating avian and swine influenza viruses and potential for H3N2 pandemic. Sci Rep. 2013;3:1822. 23661027 10.1038/srep01822 PMC3650665

[46] Broberg E, Nicoll A, Amato-Gauci A. Seroprevalence to influenza A(H1N1) 2009 virus—where are we? Clin Vaccine Immunol. 2011;18:1205–12. 21653743 10.1128/CVI.05072-11 PMC3147351

[47] Long JS, Mistry B, Haslam SM, Barclay WS. Host and viral determinants of influenza A virus species specificity. Nat Rev Microbiol. 2019;17:67–81. 30487536 10.1038/s41579-018-0115-z

[48] Borkenhagen LK, Salman MD, Ma MJ, Gray GC. Animal influenza virus infections in humans: a commentary. Int J Infect Dis. 2019;88:113–9. 31401200 10.1016/j.ijid.2019.08.002

[49] Sorrell EM, Schrauwen EJA, Linster M, De Graaf M, Herfst S, Fouchier RAM. Predicting ‘airborne’ influenza viruses: (trans-) mission impossible? Curr Opin Virol. 2011;1:635–42. 22440921 10.1016/j.coviro.2011.07.003 PMC3311991

